# The Cost-Effectiveness of Laparoscopic Adjustable Gastric Banding in the Morbidly Obese Adult Population of Australia

**DOI:** 10.1371/journal.pone.0064965

**Published:** 2013-05-22

**Authors:** Yong Yi Lee, J. Lennert Veerman, Jan J. Barendregt

**Affiliations:** University of Queensland, School of Population Health, Herston, Queensland, Australia; National Institute for Public Health and the Environment, Netherlands

## Abstract

**Background:**

To examine the cost-effectiveness of providing laparoscopic adjustable gastric banding (LAGB) surgery to all morbidly obese adults in the 2003 Australian population.

**Methods and Findings:**

Analyzed costs and benefits associated with two intervention scenarios, one providing LAGB surgery to individuals with BMI >40 and another to individuals with BMI >35, with each compared relative to a ‘do nothing’ scenario. A multi-state, multiple cohort Markov model was used to determine the cost-effectiveness of LAGB surgery over the lifetime of each cohort. All costs and health outcomes were assessed from an Australian health sector perspective and were discounted using a 3% annual rate. Uncertainty and sensitivity analyzes were conducted to test the robustness of model outcomes. Incremental cost-effectiveness ratios (ICERs) were measured in 2003 Australian dollars per disability adjusted life year (DALY) averted.

The ICER for the scenario providing LAGB surgery to all individuals with a BMI >40 was dominant [95% CI: dominant - $588] meaning that the intervention led to both improved health and cost savings. The ICER when providing surgery to those with a BMI >35 was $2 154/DALY averted [95% CI: dominant - $6 033]. Results were highly sensitive to changes in the likelihood of long-term complications.

**Conclusion:**

LAGB surgery is highly cost-effective when compared to the $50 000/DALY threshold for cost-effectiveness used in Australia. LAGB surgery also ranks highly in terms of cost-effectiveness when compared to other population-level interventions for weight loss in Australia. The results of this study are in line with other economic evaluations on LAGB surgery. This study recommends that the Australian federal government provide a full subsidy for LAGB surgery to morbidly obese Australians with a BMI >40.

## Introduction

Morbid obesity, defined as a BMI of 40 or more, is a serious condition that puts the patient at very high risk of diabetes, heart disease, and musculoskeletal disorders [1]. It is also highly resistant to dietary, lifestyle, and pharmacological interventions [2], [3], [4]. A recent meta-analysis found that diet and lifestyle modification programs lead to modest, short-term weight loss [2], with sizeable, long-term reductions in body mass limited to a small number of highly motivated individuals [4]. In contrast, randomized control trials demonstrate that bariatric surgery leads to substantive, medium-term weight loss outcomes and the resolution of co-morbidities [5].

Laparoscopic adjustable gastric banding (LAGB) presently accounts for 90% of all bariatric procedures conducted in Australia [6], and is commonly performed in Europe and South America [7], [8]. The procedure involves placing an adjustable silicon ring around the fundus of the stomach [4]. The ring regulates food intake and can be adjusted via the injection of saline through a subcutaneous access port. Recent meta-analyses show that LAGB leads to a 45–50% excess weight loss (%EWL), which is the percentage weight loss after surgery relative to the difference between pre-operative weight and an ideal weight (e.g., weight at BMI 25 kg/m^2^) [9], [10]. This corresponds with an average weight loss of 30 kg or 10 BMI units.

This study assesses the cost-effectiveness of providing LAGB to morbidly obese individuals in the adult population of Australia. It was part of the ‘ACE Prevention’ project, which examined the cost-effectiveness of 123 preventive and 27 treatment interventions in the Australian context. The study design adheres to ACE Prevention protocol [11].

## Methods

### Study framework and boundaries

This study assessed the cost-effectiveness of LAGB under a scenario where surgery is provided to all morbidly obese members of the 2003 Australian adult population. LAGB surgery was analyzed as a population-level intervention for the treatment of morbid obesity and subsequent prevention of obesity-related diseases. A health sector perspective was adopted focussing on costs and benefits accruing to patients and third-party payers (i.e., insurance companies and the government). Productivity costs were excluded as they fall outside the health sector. Patients’ time and travel costs and the cost of unrelated diseases resulting from increased life expectancy were excluded at baseline, but examined in additional costing scenarios. A discount rate of 3% per annum was applied to all costs and benefits. Health price deflators from the Australian Institute of Health and Welfare were used to adjust costs to 2003 Australian dollars [12]. Health outcomes are expressed as disability adjusted life years (DALYs). Results are reported in relation to a cost-effectiveness threshold of AU$50 000 per DALY averted [13], [14].

Current clinical guidelines by the National Institutes of Health (NIH) and National Institute for Health and Clinical Excellence (NICE) recommend weight loss surgery for individuals who have BMI >40 and failed to achieve weight loss through diet and exercise [15], [16]. People with BMI between 35 and 40 may be included if they have co-morbidities such as hypertension or diabetes. This study investigated two hypothetical scenarios. The first provided LAGB to all members of the Australian population with BMI >40, while the second extended surgery to all with BMI >35. The comparator was a ‘do nothing’ scenario where population obesity trends continue unabated. A population-level ‘diet and lifestyle’ comparator was not deemed appropriate as recipients of LAGB represent a different target population that is part of a special subset of extremely obese persons who have unsuccessfully attempted conventional weight loss methods before resorting to surgery.

### Overview of cost-effectiveness model

A multi-state, multiple cohort Markov model was constructed to calculate total health outcomes resulting from intervention weight loss in the population [17]. The multi-state life table method incorporates multiple obesity-related diseases into a life table framework and transmits disease-specific changes after weight loss into the morbidity and mortality experience of the cohort. The model partitioned the Australian population into 5-year male and female cohorts, simulating each cohort until all members died or reached 100 years of age. The intervention was assumed to be in ‘steady-state’ operation. That is, working according to its full effectiveness potential with complete availability of trained personnel and infrastructure, and the exclusion of set up costs. The model was implemented in Microsoft Excel 2007.

The nine obesity-related diseases that were modelled include: hypertensive heart disease; stroke; ischemic heart disease; diabetes mellitus; osteoarthritis; post-menopausal breast cancer; colon cancer; endometrial cancer; and kidney cancer. These were chosen based on the ‘Overweight and obesity’ chapter from the Comparative Quantification of Health Risks, which conducted systematic reviews to establish causality between high body mass and obesity-related diseases [1].

The model simulates health outcomes for two populations: (1) a reference population, with BMI distribution and disease pattern of the 2003 Australian population above age 20; and (2) the intervention population, which has identical characteristics to the reference population apart from a subset of morbidly obese persons receiving LAGB surgery. The health of both populations was simulated based on current estimates of body weight distribution and expected future trends [18]. The difference in health outcomes between the reference and intervention populations was expressed as total DALYs averted.

**Figure 1:**
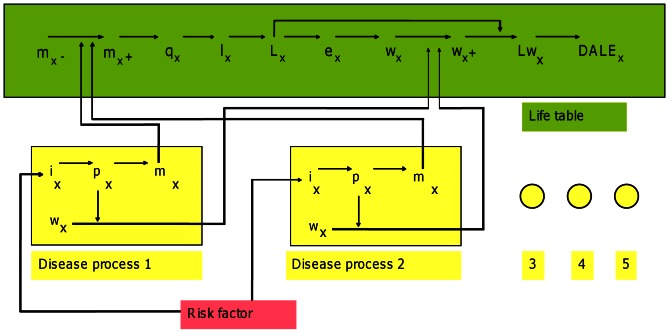
Schematic of a proportional, multi-state life table showing the interaction between disease parameters and life table parameters. In the figure: x is age; i is incidence; p is prevalence; m is mortality; w is disability-adjustment; q is probability of dying; l is number of survivors; L is life years; Lw is disability-adjusted life years; e is life expectancy and DALE is disability-adjusted life expectancy, and where ‘-’ denotes a parameter that specifically excludes modelled diseases, and ‘+’ denotes a parameter for all diseases (i.e. including modelled diseases).

A schematic overview of the model is shown in Figure 1. Health benefits were defined as increased life expectancy and improved quality of life resulting from a reduction in disease incidence following weight loss. Changes in risk factor exposure (i.e., weight loss) lead to changes in disease incidence during the same year. This leads to changes in disease prevalence at higher ages and subsequent changes to mortality. The altered mortality risks feed back into the life table and lead to changes in the number of life years lived by the cohort [17]. Changes in the prevalence of diseases modify the average quality of life across age groups. Simultaneous changes in the average quality and duration of life affect the number of disability-adjusted life years (DALYs) lived. Table 1 presents a list of the model input parameters and their sources.

**Table 1:** Description of input parameters for the cost-effectiveness model.

Input Parameters	Source
*Epidemiological inputs*	
2003 Australian population estimates	[21]
Mortality rates for the 2003 Australian population	[21]
Total pYLD rate for the 2003 Australian population	[21]
Lognormal BMI distribution for the Australian population (used to calculate BMI categories)	[24]
Incidence, prevalence, case fatality, and mortality rate for the nine obesity-related diseases	[21]
Relative risks of obesity-related disease per 1 unit increase of BMI	[1], [25]
Disability weights for obesity-related diseases	[21]
BMI trend of projected weight gain for the Australian population 20 years into the future	[46]
*Intervention inputs*	
Total weight loss following LAGB surgery	[9]
Post-operative mortality <30 days following LAGB surgery	[19]
Post-operative mortality between 30 days and 2 years following LAGB surgery	[19]
Resource use attached to initial LAGB surgery linked over two years	[27]
Annual risk of surgical maintenance two years after LAGB surgery	[20]
Annual risk of surgical complications two years after LAGB surgery	[20]
*Cost inputs*	
Cost of initial LAGB surgery linked over two years	[27]
Annual cost of surgical maintenance two years after LAGB surgery	[20]
Annual cost of surgical complications two years after LAGB surgery	[20]
Time and travel costs (hourly rate)	[11], [28]
Cost per prevalent or incident case of obesity-related disease (used to calculate cost offsets)	[12]
Health care costs for unrelated diseases and injuries due to additional years of life gained	[29]

Abbreviations: pYLD, prevalent years lived with disability; BMI, body mass index; LAGB, laparoscopic adjustable gastric banding.

### Intervention effect

A recent meta-analysis found that gastric banding surgery led to an absolute weight loss of 27.4 kg after two years and 32.0 kg [95% CI: 28.8 – 35.1] overall [7]. The model assumed that weight loss increased linearly from nothing in year zero, to 27.4 kg in year two, to a peak of 32.0 kg in year three. Weight loss was assumed to remain stable from that point onwards. This assumption was tested in a separate sensitivity analysis.

### Surgical complications

Post-operative mortality risks were taken from a meta-analysis which presents separate risks for the first 30 days post-surgery and for 30 days to 2 years post-surgery [19]. The initial 30 day mortality risk was applied to the first year of the intervention cohort after surgery, while the latter mortality risk was applied to the second year. Post-surgical mortality risk was assumed to be zero from year three onwards. Given that mortality risk after 30 days is effectively zero (based on the 30 days to 2 years mortality risk), it would be reasonable to consider the 30 day mortality risk as surgery-related mortality over the entire year. Note that this only applies for a mortality risk estimate and not a 30 day mortality rate, which would overestimate mortality attributable to surgery if applied across a year.

Rates of surgical complications were taken from a previous study examining the cost-effectiveness of LAGB in Australian diabetes patients [20]. Potential complications include increased risk of gastric prolapse, band erosion, port infection and band removal. Full details on the annual risk of post-operative mortality and complication are shown in Appendix S2.

### Modelling disease-specific impacts of weight loss surgery

The model investigated how bariatric weight loss alters disease incidence which, in turn, impacts prevalence and disease-specific mortality. Separate life tables were constructed for each obesity-related disease to calculate the prevalence and mortality of disease before and after LAGB intervention. Data on incidence, mortality, case fatality, and remission were obtained for each disease from the 2003 Australian Burden of Disease study [21]. A master life table was created to model the disability-adjusted life expectancy of the 2003 Australian cohort before and after intervention. Data on the total population, mortality, and prevalent Years Lived with Disability (pYLD) for all causes were also taken from the 2003 Australian Burden of Disease study.

The proportional multi-state life table method stipulates that the population mortality rate can be converted into ‘mortality from all other causes’ by subtracting the sum of disease-specific, pre-intervention mortality rates [17]. The disease-specific life tables calculate revised mortality rates for each disease after intervention weight loss using the Potential Impact Fraction (PIF) – an epidemiological measure that calculates the proportional change in average disease incidence after a change in exposure of a related risk factor [22], [23]. The sum of post-intervention, disease-specific mortality rates are added to the mortality rate for all other causes to determine the total mortality rate after LAGB intervention. Likewise, the disability-adjusted prevalence (i.e., pYLD) for all other causes was calculated by subtracting the sum of disease-specific pYLDs before intervention from the total pYLD attributable to all causes. The total pYLD occurring after LAGB intervention was then calculated by adding the sum of the revised pYLDs generated by disease-specific life tables. Changes in the total mortality rate and total pYLD lead to changes in the disability-adjusted life expectancy of the intervention cohort, as shown in Figure 1.

The potential impact fraction (PIF) was used to calculate the proportional change in average disease incidence after a reduction in BMI due to intervention. PIFs were calculated using three parameters: (1) prevalence of overweight and obesity in Australia; (2) relative risks of obesity-related diseases; and (3) change in body mass due to intervention.

The prevalence of overweight and obesity was calculated from lognormal distributions of BMI frequency in the Australian population partitioned by sex and age-group. Lognormal distributions were plotted based on mean and standard deviation data taken from the Australian Diabetes, Obesity and Lifestyle Study (AusDiab) [24]. Each distribution was divided into five BMI categories: (1) ‘normal weight’ or ‘under-weight’ with BMI <25; (2) ‘overweight’ with BMI 25–30; (3) ‘obese 1’ with BMI 30–35; (4) ‘obese 2’ with BMI 35–40; and (5) ‘obese 3’ with BMI >40. Proportions were calculated for each BMI category by obtaining the lognormal distribution integral between BMI cutoffs.

‘Relative risks of disease per one unit of increase in BMI’ were taken from meta-analyses conducted by the Comparative Quantification of Health Risks [1]. This was done for all diseases except diabetes, which used data from the Asia-Pacific Cohort collaboration [25]. Relative risk data are shown in Appendix S1.

The model used a modified PIF shown in Equation 1 [22]. Intervention weight loss leads to an adjustment in the relative risk of disease (RR to RR’), while the prevalence for the corresponding BMI category is held constant. RR adjustment is calculated by assuming a functional relationship between the level of risk-factor exposure and RR.
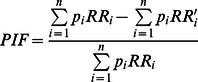
(1)


Where: p_i_ is the proportion of the population in BMI-category i; RR_i_ is the relative risk of disease associated with BMI-category i; and RR'_i_ is the relative risk of disease associated with BMI-category i after an intervention is implemented in the population.

BMI reduction leads to a change in incidence for each obesity-related disease according to Equation 2. Disease-specific life tables calculated prevalence and mortality risk before and after change in disease incidence.
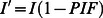
(2)


Where ***I*** is the incidence of disease (e.g. ischemic heart disease) in the population (by age and sex); ***I’*** is the new incidence of disease (e.g. ischemic heart disease) when an intervention is implemented; and ***PIF*** is the potential impact fraction for the LAGB intervention.

Each year of life lived with disability was adjusted to account for time spent in suboptimal health by assigning disease-specific disability weights shown in Appendix S1. Disability-adjusted prevalence was defined as the number of prevalent years lived with disability (pYLD) per prevalent case of disease.

The new estimates for total mortality rate and disability-adjusted prevalence were integrated in a master life table that modelled the epidemiological progression fo the 2003 Australian cohort by sex and age group. The master life table thus calculated the disability-adjusted life expectancy of the cohort resulting from intervention weight loss occurring in population members with BMI >35 and BMI >40.

### Modelling BMI trends in the Australian population

Rates of overweight and obesity have been steadily increasing in the Australian population over time [26]. The model incorporated the projected increase in future weight in Australia using results of a regression analysis by Haby & Markwick [8]. This BMI trend was applied 20 years into the future for both intervention and reference populations. Adding a trend analysis to the model is expected to increase the effectiveness of the intervention and make LAGB surgery slightly more cost-effective than if these trends were ignored.

### Overview of costing scenarios

This study examined three costing scenarios. The baseline scenario adopted a health care perspective with focus on direct costs accruing to the government, patients and health insurers. This scenario included cost offsets arising from reduced incidence of obesity-related disease after intervention. The second scenario additionally included patients’ time and travel costs resulting from intervention. In the third scenario the cost of unrelated diseases due to additional years of life patients acquire after intervention was added.

### Intervention costs

Estimates on the cost of the initial intervention, maintenance, and ensuing complications were sourced from two previous studies [20], [27]. Additional data were obtained through personal correspondence with study authors. Table 2 provides a brief summary of costs associated with LAGB intervention. The cost of the initial intervention, which includes two-year follow up, was applied to the subset of the intervention cohort receiving LAGB surgery. The aggregate annual cost of maintenance and complication was applied to surviving members of the subset from year three onwards. Detailed information on intervention costs and resource inputs are shown in Appendix S2.

**Table 2:** Costs associated with LAGB intervention for two different scenarios (2003 AU$).

Costing item	Cost/person
*Baseline scenario*	
Cost of initial LAGB surgery	11 290
Annual cost of maintenance	330
Annual cost of complications	90
*Scenario including time & travel costs*	
Cost of initial LAGB surgery	12 452
Annual cost of maintenance	1 126
Annual cost of complications	105

### Time and travel costs

Patients’ time was valued at 25% of the hourly wage rate and calculated based on the proportion of the Australian population employed, unemployed or not part of the workforce. The overall time cost, which applied universally across age and sex, amounted to $17.44 per hour [11]. A cost of $9.39 was used for travel to and from the hospital and was taken from another hospital-based study in Australia [28]. Travel costs for outpatient, specialist and GP visits were assumed to be identical. Detailed information on time spent in hospital and outpatient services is shown in Appendix S2.

### Disease costs

Intervention costs were partially offset by reductions in health expenditure due to lower incidence of diseases linked to excess body weight. Treatment costs were obtained from the Australian Institute of Health and Welfare Disease Costs and Impacts Study 2001 (see Appendix S2) [29]. The average cost offset was calculated for each incident case averted in relation to cancer (i.e., colon cancer, breast cancer, endometrial cancer and kidney cancer) and each prevalent case averted for remaining diseases. A one–off cost per incident case was applied to the four cancers as prevalence was difficult to define and costs were largely clustered around the incident event. Cost offsets were based on rates of disease during 2001 [12]. Health care costs for unrelated diseases and injuries due to additional years of life gained were also taken from the Disease Costs and Impacts Study [29].

### Sensitivity and uncertainty analysis

Sensitivity analysis was used to test the robustness of model assumptions. Discount rates of 0% and 6% were used to compare results with those using the baseline rate of 3%. The annual risk of each post-surgical complication was perceived to be small, so a joint sensitivity analysis was conducted by increasing the annual probability for each complication by one order of magnitude. An additional sensitivity analysis altered the stable weight loss assumption. Data from O’Brien et al.[30] was used to calculate the annual percentage attenuation of weight loss outcomes in the 15 years following LAGB surgery (see Appendix S1). Weight loss was assumed to remain stable after 15 years.

Uncertainty analysis was conducted to assess the level of parameter uncertainty and its effect on final cost-effectiveness results. Monte Carlo simulations were implemented using the Excel add-in Ersatz (www.epigear.com) to obtain 95% uncertainty intervals for DALY’s averted, net costs and ICERs. Simulations were based on 3 000 iterations. Table 3 presents the input uncertainty parameters and their distributions.

**Table 3:** List of uncertainty parameters and associated distributions.

Uncertainty Parameter	Value (uncertainty range)	Uncertainty distribution	Source
*Intervention inputs*			
Weight loss after LAGB surgery (kg)	–31.97 (SE 1.62)	Normal	[9]
RR of disease per 1 unit increase of BMI	See Appendix S1	Relative risk	[1], [25]
*Risk of complications <2 yrs after initial surgery*			
Lap-band removal and replacement	0.07 (N = 30)	Beta	[27]
Hospital admission due to port infection	0.03 (N = 30)	Beta	[27]
Lap-band removal	0.03 (N = 30)	Beta	[27]
*Frequency of outpatient consultations <2 yrs after initial surgery*			
Surgeon/physician	11.60 (SE 4.10)	Gamma	[27]
Surgeon/physician + lap-band adjustment	10.27 (SE 4.58)	Gamma	[27]
Respiratory physician	0.43 (SE 1.14)	Gamma	[27]
Dietician	0.10 (SE 0.31)	Gamma	[27]
*Post-surgical mortality risk*			
Risk of death <30 days	0.0006 (SE 0.0003)	Gamma	[19]
Risk of death >30 days to 2 years	0.0000 (SE 0.0002)	Gamma	[19]
*Annual risk of long-term surgical complications*			
Gastric prolapse	0.01 (N = 1120)	Beta	[20]
Erosion of band into the stomach	0.001 (N = 1120)	Beta	[20]
Port infection	0.002 (N = 1120)	Beta	[20]
Band removal	0.004 (N = 1120)	Beta	[20]

Abbreviations: SE, standard error; N, sample size; LAGB, laparoscopic adjustable gastric banding; RR, relative risk; BMI, body mass index.

### Additional analyses to facilitate government decision making

Descriptive analyses were conducted to examine the LAGB intervention in light of its ‘strength of evidence’, ‘feasibility’, ‘acceptability to stakeholders’, ‘impact on inequalities’, ‘sustainability’ and ‘relevance to the indigenous population’. A simple budget impact analysis was also conducted to gauge the fiscal impact of publicly funding LAGB surgery in Australia during the first year of implementation.

## Results

### Results of the baseline scenario

Results of the baseline analysis are presented in Table 4. When provided to all Australians with BMI >40, the LAGB intervention improves health while simultaneously achieving net cost savings and is therefore *dominant* [95% UI: *dominant* – $588]. When LAGB surgery is extended to all Australians with BMI >35, the mean ICER becomes $2 154/DALY averted [95% UI: *dominant* –$6 033]. Under this scenario health gains and cost offsets increase 3-fold while the cost of intervention, maintenance and complications increases 5-fold, compared to the scenario in which LAGB surgery is limited to individuals with BMI >40.

**Table 4:** Summary of cost-effectiveness results for the two baseline scenarios (2003 AU$).

	Surgery for BMI >40	Surgery for BMI >35
**Total intervention cost**		
*Mean*	$1 590 m	$8 075 m
*(95% UI)*	($1 433 m – $1 797 m)	($7 288 m – $9 173 m)
**Total cost of maintenance & complications**		
*Mean*	$1 028 m	$5 313 m
*(95% UI)*	($931 m – $1 148 m)	($4 843 m – $5 946 m)
**Total cost offsets**		
*Mean*	-$3 737 m	-$11 067 m
*(95% UI)*	( -$5 753 m – -$2 505 m)	( -$14 699 m – -$8 281 m)
**Total net costs**		
*Mean*	–$1 119 m	$2 322 m
*(95% UI)*	( –$3 116 m – $160 m)	( –$1 566 m – $5 438 m)
**Total DALYs averted**		
*Mean*	441 749	1 250 067
*(95% UI)*	*(266 366 – 735 079)*	*(874 398 – 1 755 167)*
**ICER**		
*Mean*	dominant	$2 154
*(95% UI)*	(dominant – $588)	(dominant –$6 033)

Abbreviations: LAGB, laparoscopic adjustable gastric banding; BMI, body mass index; ICER, incremental cost-effectiveness ratio; 95% UI, 95% uncertainty interval.

Note: Total intervention costs relate to the cost of initial LAGB surgery and post-surgical follow-up. Total cost of maintenance and complications includes all surgical and outpatient costs during the remainder of a LAGB recipient’s life. Total cost offsets include all health care costs avoided due to lower risk of obesity-related disease after surgery. Total net costs are the aggregate sum of the total intervention cost, the cost of maintenance and complications, and all cost offsets. Total DALYs averted denotes the incremental benefit of the LAGB intervention relative to the status quo.

There is a 94.5% probability of LAGB surgery being cost-saving when applied to people with BMI >40. This becomes 10.1% when extended to those with BMI >35. In both cases, there is a 100% probability that the ICER is less than $10 000/DALY averted.

### Results of the sensitivity analysis and costing scenarios

The results of the three univariate sensitivity analyses and the three costing scenarios are shown in Table 5 (BMI >40) and Table 6 (BMI >35). Baseline ICERs did not change dramatically when using a 0% discount rate, including patients’ time and travel costs, including cost of unrelated diseases, or attenuating the peak weight loss over 15 years post-surgery. A 6% discount rate made little difference when LAGB surgery is limited to people with a BMI >40, but led to a substantially higher ICER when surgery was extended to people with BMI >35. For both the BMI >35 and the BMI >40 scenarios, the largest observed changes to baseline ICERs occurred after: excluding cost offsets; and increasing the rates of maintenance and complication by one order of magnitude. Much of the change in the latter scenario occurred from a 3-fold increase in the total cost of maintenance and complications (data not shown). After increasing rates of maintenance and complications, the probability of the ICER lying below $50 000/DALY became 99.5% for BMI >40 and 94.4% for BMI >35.

**Table 5:** Sensitivity analysis for intervention population with BMI >40 (2003 AU$).

Scenario	Total net costs		Total DALYs averted		ICER	
	*Mean*	*(95% UI)*	*Mean*	*(95% UI)*	*Mean*	*(95% UI)*
Baseline	–$1 119 m	(–$3 116 m – $160 m)	441 749	(266 366 – 735 079)	dominant	(dominant – $588)
1a) 0% discount rate	–$5 476 m	(–$10 508 m – $2 391 m)	1 222 382	(741 701 – 2 036 792)	dominant	(dominant – dominant)
1b) 6% discount rate	$434 m	(–$573 m – $1 056 m)	192 195	(118 956 – 325 582)	$2 913	(dominant – $8 738)
2) Including time & travel costs	–$659 m	(–$2 633 m – $625 m)	Identical to baseline scenario		dominant	(dominant – $2 280)
3) Excluding cost offsets	$2 618 m	($2 421 m – $2 851 m)	Identical to baseline scenario		$6 329	($3542 – $9885)
4) Including cost of unrelated diseases	$1 107 m	(–$81 m – $1 835 m)	Identical to baseline scenario		$2 925	(dominant – $6 638)
5) Higher rates of complication	$2 502 m	($468 m – $4 445 m)	250 811	(110 869 – 466 283)	$12 851	($1 036 – $37 413)
6) Weight loss attenuation over 15 years	–$880 m	(–$2 796 m – $341 m)	419 944	(258 460 – 703 113)	dominant	(dominant – $1 249)

Abbreviations: LAGB, laparoscopic adjustable gastric banding; BMI, body mass index; ICER, incremental cost effectiveness ratio; 95% UI, 95% uncertainty interval.

Note: Estimates ‘identical to baseline’ occur as these scenarios were simultaneously analyzed with the baseline scenario. The alternate scenarios piggyback the results of the baseline analysis.

**Table 6:** Sensitivity analysis for intervention population with BMI >35 (2003 AU$).

Scenario	Total net costs		Total DALYs averted		ICER	
	*Mean*	*(95% UI)*	*Mean*	*(95% UI)*	*Mean*	*(95% UI)*
Baseline	$2 322 m	(–$1 566 m – $5 438 m)	1 250 067	(874 398 – 1 755 167)	$2 154	(dominant –$6 033)
1a) 0% discount rate	–$8 548 m	(–$16 929 m – –$1 732 m)	3 432 824	(2 445 799 – 4 802 291)	dominant	(dominant – dominant)
1b) 6% discount rate	$6 120 m	($4 165 m – $7 985 m)	553 239	(386 043 – 781 280)	$11 673	($5 442 – $20 146)
2) Including time & travel costs	$4 679 m	($728 m – $7 874 m)	Identical to baseline scenario		$4 102	($417 – $8 720)
3) Excluding cost offsets	$13 389 m	($12 388 m – $14 627 m)	Identical to baseline scenario		$11 069	($7 473 – $15 527)
4) Including cost of unrelated diseases	$9 082 m	($6 780 m – $11 084 m)	Identical to baseline scenario		$7 624	($4 009 – $12 102)
5) Higher rates of complication	$17 787 m	($10 535 m – $26 378m)	715 135	(369 096 – 1 190 195 )	$28 692	($9 278 – $65 973)
6) Weight loss attenuation over 15 years	$3 156 m	(–$388 m – $6 127 m)	1 172 303	(811 172 – 1 651 019)	$3 004	($811 – $1 651)

Abbreviations: LAGB, laparoscopic adjustable gastric banding; BMI, body mass index; ICER, incremental cost effectiveness ratio; 95% UI, 95% uncertainty interval.

Note: Estimates ‘identical to baseline’ occur as these scenarios were simultaneously analyzed with the baseline scenario. The alternate scenarios piggyback the results of the baseline analysis.

### Budget impact analysis

Under the budget impact analysis, 140 673 Australians had a BMI >40 in the year 2003, while 714 821 had a BMI >35. LAGB surgery cost $11 290 per person. If the government was to fund LAGB surgery for all Australians with BMI >40 (and all eligible persons underwent surgery within a year) then the total cost would be $1 588 million. If surgery were extended to all Australians with BMI >35 then this would cost $8 070 million. The portion of total health expenditures funded by the Australian government in the years 2003-04 amounted to $35 729 million [31]. Fully subsidizing LAGB surgery for all Australians with BMI >40 would thus constitute a one-off 4.4% increase to all health expenditures funded by the Australian government in 2003. This would be an additional 22.6% if LAGB surgery were extended to those with BMI >35.

## Discussion

### Interpretation of results

LAGB surgery is a cost-effective, albeit expensive intervention for obesity. The results of this study demonstrate that LAGB surgery is cost-saving when provided to all Australians with BMI >40, and highly cost-effective compared to a $50 000/DALY threshold when extended to people with BMI >35. LAGB surgery continues to have a high probability of being cost-effective even with a 10-fold increased risk of complications.

The results of this study are in line with previous economic evaluations on LAGB surgery [20], [32], [33], [34], [35], [36], [37], [38], [39]. Campbell et al.[35] used a lifetime Markov model to examine the cost-effectiveness of LAGB surgery for individuals in the U.S. aged 18-75 years with BMI >35, resulting in an ICER of $5 400/QALY (2005 US$). Salem et al.[38] used a deterministic, decision analytic model over a 3 year horizon on men and women in the U.S. aged 35 years with a BMI >40. They calculated an ICER of $11 604/QALY for men and $8 878/QALY for women (2004 US$). The shorter time horizon may explain the higher ICERs as health benefits from reduced body mass don’t accrue until later in life. Two studies from the U.K. found that LAGB surgery led to an ICER well below the £30 000/QALY cost-effectiveness threshold used by NICE [36], [37].

Two studies have been conducted in the Australian context [20], [33]. The first used a lifetime Markov model to determine cost-effectiveness in a cohort of Australian adolescents (aged between 14-19 years) who had a BMI >35 [33]. The comparator was current practice. The authors calculated an ICER of $4 400/DALY [95% UI: 2 900 – 6 120] in 2001 Australian dollars. The second study examined the cost-effectiveness of LAGB surgery as a means of resolving co-morbidities associated with type 2 diabetes [20]. Patient-level data were used to compare the effectiveness of LAGB surgery to conventional diabetes therapy. The authors of this study examined results in 2006 Australian dollars per QALY and found an overall ICER that was *dominant* [95% UI: *dominant* – 48 400]. These results are in line with other international studies, which have also found that LAGB surgery dominates conventional diabetes therapy [32], [34].

### Additional policy considerations

Beyond considerations of efficiency and cost-effectiveness, it is expected that the provision of LAGB surgery as a population-wide health intervention will be hindered by: (1) pejorative attitudes towards obese persons held by members of society [40]; (2) the substantial upfront cost of surgery; and (3) limited capacity in the short-run to immediately provide lap bands and surgery to the entire intervention population.

### Study strengths and limitations

This study is the first Australian economic evaluation on the cost-effectiveness of implementing LAGB surgery in the morbidly obese adult population of Australia. The results have been tailored towards facilitating budget allocation decisions by policy makers in the Australian health sector. One of the biggest strengths of this study, which differentiates it from the existing literature, is its inclusion in the broader ACE Prevention framework. The use of common evaluation methods means that the results of this analysis are potentially comparable with the results of other interventions assessed under ACE Prevention. LAGB surgery was ranked among the most cost-effective preventive interventions with the largest population health impact in ACE Prevention. This list also included tobacco taxation, alcohol taxation, and the polypill to lower blood pressure and cholesterol [11].

**Figure 2:**
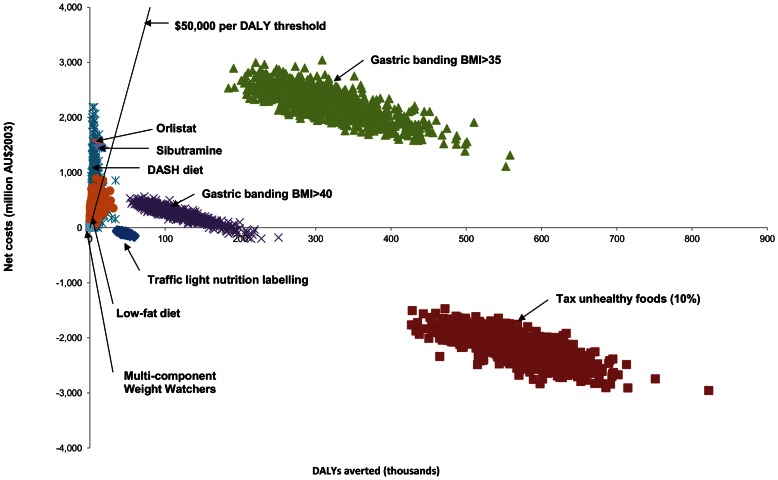
Cost-effectiveness scatterplot of high body mass interventions analyzed under ACE Prevention. Note that all interventions were analyzed with the inclusion of time and travel costs.

Figure 2 presents a scatterplot of cost-effectiveness results for the different high body mass interventions analyzed under ACE Prevention. Two policy-based interventions which aim to prevent diseases associated with high body mass were also found to be cost-saving under ACE Prevention. The first was a 10% tax on unhealthy food and the second was a ‘traffic light’ nutrition labeling intervention (which briefly involves placing a green light on the packaging of healthy food and a red light on unhealthy food) [41]. While the strength of evidence for these interventions is considered weaker, the upfront cost of these interventions is also much lower than that for bariatric surgery. The upfront cost of implementing the 10% ‘unhealthy food’ tax and the ‘traffic light’ intervention was estimated at $18 million and $81 million respectively, which is a fraction of the cost to provide LAGB surgery to all adult Australians with BMI >40. In addition, these interventions target the entire population rather than focusing solely on the morbidly obese. It is expected that the 10% tax and ‘traffic light’ interventions may be more politically palatable than subsidising LAGB surgery on a widespread basis. However, such regulatory interventions would face strong resistance from the food industry.

One notable limitation is the conflicting evidence on the long-term consequences of LAGB surgery beyond 5 years. Rates of maintenance and complications were based on the best available Australian data at the time of study. However, uncertainty remains as to whether low rates of complication extend beyond the short- to medium-term. A long-term study by Carelli et al.[42] retrospectively followed 2 909 patients who underwent LAGB surgery at the New York University hospital between 2001 and 2008. Non-device related complications occurred in 2.06% of patients. The highest rate of device related complication was slippage/prolapse (4.52%), while the lowest was band erosion (0.24%). A study by Favretti et al.[43] examined 1 791 consecutive LAGB patients in an Italian hospital, with up to 12 years follow up. Major complications requiring re-operation occurred in 5.9% of patients, while minor complications occurred in 11.2%. Lanthaler et al.[44] followed up 276 patients for 9 years, with 52.9% of patients having at least one complication requiring reoperation. Likewise, Suter et al.[45] followed up 317 patients for a total of 8 years. Late complications such as band erosion and pouch dilation/slippage occurred in 33.1% of patients, with 21.7% requiring major re-operation. It is difficult to synthesize such conflicting data. If rates of complication are much higher than estimated in this study, then it is likely that the LAGB intervention will not be as cost-effective as initially thought. Furthermore, this analysis is unable to provide information on the potential for other consequences such as psychiatric outcomes.

Uncertainty information was not available for several important input parameters which are not reflected in the final ICER uncertainty intervals. These include: disease disability weights; annual risk/cost of long-term surgical maintenance 2 years after initial LAGB surgery; cost offset data; health care costs for unrelated diseases and injuries; and time and travel costs.

In reality, it is highly unlikely that every morbidly obese individual will receive bariatric surgery. Current clinical guidelines state that bariatric surgery is an intervention of last resort for morbidly obese people who have exhausted all other means of achieving weight loss. As no information was available on the uptake rate of subsidised LAGB surgery, this analysis assumed that all morbidly obese individuals would receive the intervention. However, this assumption will not affect the final results regarding cost-effectiveness. For example, if a participation rate of 50% or 70% of the morbidly obese population were modelled then the costs and benefits accrued would simply equate to 50% and 70% respectively and the final cost-effectiveness ratio would not change.

The model was conducted under ‘steady-state’ conditions, which assumes that the intervention achieves its effectiveness potential with fully available capital resources and no set up costs. It is unreasonable to expect that there will be enough available surgeons, hospital staff, and facilities to provide LAGB surgery on a population basis. Prioritisation rules such as ‘clinical urgency’ may need to be implemented to determine who should receive LAGB surgery in the face of limited resources. This was not investigated in the current study and is an area requiring further research.

### Policy recommendation

Australians are currently eligible for government rebates on LAGB surgery, as listed on the Medical Benefits Scheme (MBS). However, the current $802.90 rebate is paltry compared to the overall cost of surgery (∼$13 000). This study found that LAGB surgery is a dominant intervention when limited to individuals with a BMI >40 over a lifetime horizon. This leads to both improved health and overall cost savings, such that reductions in future expenditure on obesity-related diseases outweigh the initial cost of surgery. From these results, it is recommended that the Australian government begin fully subsidising LAGB surgery for all Australians with a BMI >40. Providing a full subsidy to eligible individuals with a BMI between 35 and 40 would not be advisable as this would potentially lead to an unsustainable increase in government expenditures. However, caution should be applied as restricting subsidies to those with BMI >40 may lead to moral hazard – where individuals with a high BMI just below 40 have an incentive to gain extra weight to obtain subsidy.

The results of this study may have some relevance to other high income OECD countries like Canada and the United Kingdom, which face similar contributing factors for their obese populations (i.e., poor diet and lifestyle, obesogenic environments, pressure from food industry lobby groups) and have publicly funded health systems akin to Australia’s. However, cost-effectiveness analysis is a locally bound activity. Recommendations for the reimbursement of LAGB surgery in these countries should be based on locally obtained data that reflect their respective context.

## Conclusion

This study found that LAGB surgery was cost-saving when provided to all individuals with a BMI >40. LAGB surgery was also cost-effective when extended to all individuals with a BMI >35, but at a substantial aggregate cost. This study recommends that the Australian government consider providing a full subsidy on LAGB surgery for all Australians with a BMI >40.

## Supporting Information

Appendix S1 **Epidemiological input parameters.**
(DOCX)

Appendix S2 **Cost data and sources.**
(DOCX)
