# Correction: The Cost-Effectiveness of Laparoscopic Adjustable Gastric Banding in the Morbidly Obese Adult Population of Australia

**DOI:** 10.1371/journal.pone.0106266

**Published:** 2014-08-15

**Authors:** The PLOS ONE Staff

Figure 2 and its legend are incorrect. The authors have provided the correct Figure 2 and its legend here.

**Figure 2:**
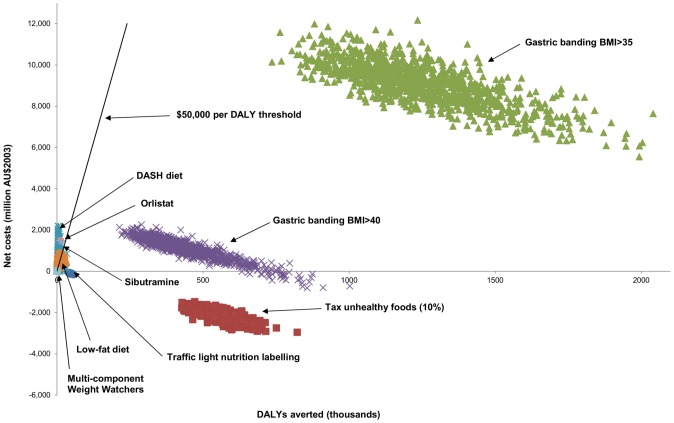
Cost-effectiveness scatterplot of high body mass interventions analyzed under ACE Prevention. Note that all interventions were analyzed with the inclusion of time and travel costs and the cost of unrelated diseases.

## References

[1] Lee YY, Veerman JL, Barendregt JJ (2013) The Cost-Effectiveness of Laparoscopic Adjustable Gastric Banding in the Morbidly Obese Adult Population of Australia. PLoS ONE 8(5): e64965 doi:10.1371/journal.pone.0064965 23717680 10.1371/journal.pone.0064965 PMC3661518

