# Supplementary material for: The Cost-Effectiveness of Laparoscopic Adjustable Gastric Banding in the Morbidly Obese Adult Population of Australia
Source: PLoS One. 2013 May 22;8(5):e64965. doi: 10.1371/journal.pone.0064965 (PMC3661518; doi:10.1371/journal.pone.0064965)
Supplement: Appendix S1 — Epidemiological input parameters. (DOCX) [file pone.0064965.s001.docx]

## Appendix S1: Epidemiological input parameters

**Table 1.** Relative risks of disease per 1 unit increase of BMI.

**Table 2.** Disability weights for prevalent diseases, by sex, at baseline.

**Table 3.** Percentage attenuation of peak weight gain during 15 years following LAGB surgery.

**Table 1.** Relative risks of disease per 1 unit increase of BMI [1,2].

|  | **Age** | **Males** | **Females** |
| --- | --- | --- | --- |
| Colorectal cancer | <35 | 1 | 1 |
|  | 35+ | 1.03 (1.01-1.05) | 1.03 (1.01-1.05) |
| Breast cancer | <35 | - | 1 |
|  | 35+ | - | 1.03 (1.02-1.04) |
| Endometrial cancer | <35 | - | 1.10 (1.07-1.14) |
|  | 35+ | - | 1.10 (1.07-1.14) |
| Kidney cancer | <35 | 1.06 (1.03-1.08) | 1.06 (1.03-1.08) |
|  | 35+ | 1.06 (1.03-1.08) | 1.06 (1.03-1.08) |
| Osteoarthritis | <35 | 1.04 (1.03-1.06) | 1.04 (1.03-1.06) |
|  | 35+ | 1.04 (1.03-1.06) | 1.04 (1.03-1.06) |
| Ischemic heart disease | <35 | 1 | 1 |
|  | 35-44 | 1.12 (1.05-1.19) | 1.12 (1.05-1.19) |
|  | 45-59 | 1.10 (1.08-1.14) | 1.10 (1.08-1.14) |
|  | 60-69 | 1.06 (1.03-1.08) | 1.06 (1.03-1.08) |
|  | 70-79 | 1.04 (1.02-1.06) | 1.04 (1.02-1.06) |
|  | 80+ | 1.02 (1.00-1.05) | 1.02 (1.00-1.05) |
| Hypertensive heart disease | <45 | 1 | 1 |
|  | 45-59 | 1.09 (1.03-1.14) | 1.09 (1.03-1.14) |
|  | 60-69 | 1.16 (1.05-1.27) | 1.16 (1.05-1.27) |
|  | 70-79 | 1.12 (1.04-1.21) | 1.12 (1.04-1.21) |
|  | 80+ | 1.06 (1.02-1.11) | 1.06 (1.02-1.11) |
| Stroke | <35 | 1 | 1 |
|  | 35-44 | 1.14 (1.05-1.23) | 1.14 (1.05-1.23) |
|  | 45-59 | 1.10 (1.03-1.16) | 1.10 (1.03-1.16) |
|  | 60-69 | 1.08 (1.03-1.13) | 1.08 (1.03-1.13) |
|  | 70-79 | 1.05 (1.02-1.09) | 1.05 (1.02-1.09) |
|  | 80+ | 1.03 (1.01-1.05) | 1.03 (1.01-1.05) |
| Type II Diabetes | <35 | 1 | 1 |
|  | 35-44 | 1.19 (1.06-1.32) | 1.19 (1.06-1.32) |
|  | 45-69 | 1.14 (1.05-1.23) | 1.14 (1.05-1.23) |
|  | 70+ | 1.10 (1.03-1.16) | 1.10 (1.03-1.16) |
| NB. Values shown are the mean and 95% confidence intervals. | | | |

**Table 2.** Disability weights for prevalent diseases, by sex, at baseline [3].

|  | Male | Female |
| --- | --- | --- |
| Colorectal Cancer | 0.12 | 0.11 |
| Breast cancer | - | 0.12 |
| Endometrial cancer | - | 0.03 |
| Kidney cancer* | 0.06 | 0.06 |
| Ischemic heart disease* | 0.04 | 0.06 |
| Stroke* | 0.31 | 0.31 |
| Hypertensive heart disease | 0.09 | 0.07 |
| Type II Diabetes* | 0.08 | 0.08 |
| Osteoarthritis* | 0.05 | 0.06 |

* Disability weights used differ by age; weighted average at baseline (2003) is presented.

**Table 3.** Percentage attenuation of peak weight gain during 15 years following LAGB surgery [4].

|  | Percentage attenuation of weight loss |
| --- | --- |
| Year 1 | 100% |
| Year 2 | 100% |
| Year 3 | 100% |
| Year 4 | 100% |
| Year 5 | 98% |
| Year 6 | 95% |
| Year 7 | 95% |
| Year 8 | 95% |
| Year 9 | 92% |
| Year 10 | 93% |
| Year 11 | 91% |
| Year 12 | 94% |
| Year 13 | 93% |
| Year 14 | 96% |
| Year 15 | 93% |

N.B. Peak weight loss occurs at year 3 in the model, after which progressive weight loss attenuation begins until year 15.

## Reference List

1. James WP, Jackson-Leach R, Ni Mhurchu C, Kalamara E, Shayeghi M, et al. (2004) Overweight and obesity (high body mass index). In: Ezzati M, Lopez AD, Rodgers A, Murray CJL, editors. Comparative quantification of health risks. Geneva: World Health Organization. pp. 497-596.

2. Ni Mhurchu C, Parag V, Nakamura M, Patel A, Rodgers A, et al. (2006) Body mass index and risk of diabetes mellitus in the Asia-Pacific region. Asia Pac J Clin Nutr 15: 127-133.

3. Begg S, Vos T, Barker B, Stevenson C, Stanley L, et al. (2007) The burden of disease and injury in Australia 2003. Canberra: AIHW. PHE 82 PHE 82.

4. O'Brien PE, MacDonald L, Anderson M, Brennan L, Brown WA (2013) Long-term outcomes after bariatric surgery: fifteen-year follow-up of adjustable gastric banding and a systematic review of the bariatric surgical literature. Ann Surg 257: 87-94.
