# Supplementary material for: The Cost-Effectiveness of Laparoscopic Adjustable Gastric Banding in the Morbidly Obese Adult Population of Australia
Source: PLoS One. 2013 May 22;8(5):e64965. doi: 10.1371/journal.pone.0064965 (PMC3661518; doi:10.1371/journal.pone.0064965)
Supplement: Appendix S2 — Cost data and sources. (DOCX) [file pone.0064965.s002.docx]

## Appendix S2: Cost data and sources

**Table 1.** Mean resource use (per patient) observed over two years for persons undergoing LAGB surgery.

**Table 2.** Annual probability and cost (per person) associated with post-operative mortality, surgical maintenance and complication.

**Table 3.** A summary of the average time spent in hospital and outpatient services during the intervention.

**Table 4.** Cost per prevalent or incident case of disease.

**Table 1.** Mean resource use (per patient) observed over two years for persons undergoing LAGB surgery [1].

| **Cost category** | **Inclusions** | **Source** | **Mean resource use per patient** | **Standard Deviation** | **Unit cost (2003 AU$)** | **Mean cost per patient (2003 AUD)** |
| --- | --- | --- | --- | --- | --- | --- |
| *LAGB surgery (private hospital)* | Specialist medical personnel (surgeon, surgical assistant, or anesthetist | [1] | 0.97 | 0.18 | 2,749 | 7,814 |
|  | Hospital personnel costs | [1] | 0.97 | 0.18 | 827 |  |
|  | LAGB prosthesis | [1] | 0.97 | 0.18 | 2,991 |  |
|  | Theatre supplies, non-theatre supplies, and other expenses | [1] | 0.97 | 0.18 | 1,516 |  |
| *Mitigation of surgical complications* | Lap-band removal and replacement | [1] | 0.07 | 0.25 | 8,084 | 794 |
|  | Hospital admission due to port infection | [1] | 0.03 | 0.18 | 2,548 |  |
|  | Lap-band removal | [1] | 0.03 | 0.18 | 5,092 |  |
| *Outpatient medical consultations* | Surgeon/physician | MBS item 23 | 11.60 | 4.10 | 30.20 | 1,515 |
|  | Surgeon/physician + lap-band adjustment | MBS items 23 & 14215 | 10.27 | 4.58 | 111.55 |  |
|  | Respiratory physician | MBS item 105 | 0.43 | 1.14 | 35.65 |  |
|  | Dietician | [1] | 0.10 | 0.31 | 36.66 |  |
| *Outpatient medical pathology tests* | Not described | [1] |  | * |  | 579 |
| *Outpatient medical investigations* | Barium meal | MBS item 58909 | 1.00 | – | 84.80 | 588 |
|  | Sleep study | MBS item 12203 | 1.03 | – | 488.65 |  |
| *Total* |  |  |  |  |  | *11,290* |

NOTE: This table summarizes the mean resource use per patient from a clinical sample of 30 patients over a two year period. All cost data taken from Keating et al.[9] were originally quoted in 2006 AU$. These prices were converted in 2003 AU$ by using an appropriate AIHW health price deflator. When possible, the 2003 price of Medicare Benefits Scheme (MBS) items was obtained rather than converting 2006 MBS items into 2003 AU$

* Outpatient medical pathology tests (16 tests) were measured in detail but excluded from the table due to extensive detail.

**Table 2.** Annual probability and cost (per person) associated with post-operative mortality, surgical maintenance and complication.

|  | **Annual units / Probability** | **Unit Cost (2003 AU$)** | **Annual Cost (2003 AU$)** | **Source** |
| --- | --- | --- | --- | --- |
| **Post-surgical mortality rates** |  |  |  |  |
| Death ≤ 30 days | 0.0006 | N/A | N/A | [2] |
| Death > 30 days to 2 years | 0.0000 | N/A | N/A | [2] |
|  |  |  |  |  |
| **Surgical therapy maintenance (surgical patients)** |  |  |  |  |
| *Outpatient medical consultations* |  |  |  |  |
| Surgeon/physician/GP | 2 | 29.32 | 58.65 | [3] |
| Surgeon/physician/GP + lap band adjustment | 2 | 109.05 | 218.10 | [3] |
| *Medical investigations* |  |  |  |  |
| Barium meal (tests) | 0.3 | 82.48 | 24.74 | [3] |
| Gastroscopy (investigations) | 0.2 | 143.87 | 28.77 | [3] |
|  |  |  |  |  |
| **Surgical therapy complications (events)** |  |  |  |  |
| Gastric prolapse* | 0.01 | 5,277 | 52.77 | [3] |
| Erosion of the band into the stomach* | 0.001 | 13,463 | 13.46 | [3] |
| Port infection* | 0.002 | 2,470 | 4.94 | [3] |
| Band removal | 0.004 | 4,705 | 18.82 | [3] |

* In the case of gastric prolapse and port infection, the unit cost is the sum of the cost associated with the event plus the cost of removal and replacement of the lap-band. In the case of band erosion, it is assumed that the band is removed entirely with no replacement and that the patient reverts back to their original pre-intervention weight after one Markov cycle (i.e., one year).

**Table 3.** A summary of the average time spent in hospital and outpatient services during the intervention.

| **Intervention component** | **Time (Hours)** | **Source** |
| --- | --- | --- |
| Initial LAGB surgery at baseline | 31.6 | [1] |
| Complications |  |  |
| *Repeat LAGB* | 31.9 | [3] |
| *Prolapse* | 31.9 | [3] |
| *Erosion (two admissions)* | 63.8 | [3] |
| *Port Infection Surgery (4 day admission to public hospital)* | 96.5 | [3] |
| *Explantation* | 24.5 | [3] |
| Time spent during outpatient visit with a specialist or GP | 0.75 | Author’s estimate |
| Sleep study | 12 | Author’s estimate |

NOTE: Patient time was valued at an average cost of $17.44 per hour for all ages and sex [4]. Travel to and from the hospital/outpatient services was valued at $9.39 [5]. Costs are in 2003 Australian dollars.

**Table 4.** Cost per prevalent or incident case of disease [6].

| **Age** | **Colon Cancer*** | **Breast Cancer*** | **Endo-metrial Cancer*** | **Kidney Cancer*** | **Ischemic Heart Disease**** | **Stroke**** | **Hypertensive Heart Disease**** | **Type II Diabetes**** | **Osteo-arthritis**** | **All other***** |
| --- | --- | --- | --- | --- | --- | --- | --- | --- | --- | --- |
| Males |  |  |  |  |  |  |  |  |  |  |
| <55 | $17,490 | – | – | $16,298 | $2,962 | $2,228 | $13,103 | $504 | $4,431 | $1,555 |
| 55–64 | $17,657 | – | – | $16,751 | $1,988 | $4,942 | $24,408 | $660 | $4,431 | $2,828 |
| 65–74 | $18,164 | – | – | $14,748 | $1,664 | $9,529 | $15,048 | $763 | $4,431 | $4,731 |
| 75-84 | $18,037 | – | – | $14,526 | $1,512 | $12,856 | $8,167 | $639 | $4,431 | $7,945 |
| 85+ | $19,288 | – | – | $7,372 | $1,394 | $16,301 | $1,723 | $594 | $4,431 | $13,061 |
| Females |  |  |  |  |  |  |  |  |  |  |
| <55 | $17,136 | $12,424 | $10,665 | $15,505 | $1,832 | $1,161 | $22,097 | $506 | $4,431 | $2,009 |
| 55–64 | $16,349 | $10,493 | $9,902 | $16,363 | $1,520 | $2,090 | $32,044 | $759 | $4,431 | $3,225 |
| 65–74 | $17,238 | $11,609 | $14,419 | $17,133 | $1,595 | $5,106 | $20,357 | $839 | $4,431 | $4,829 |
| 75-84 | $17,360 | $12,706 | $10,497 | $17,198 | $1,564 | $13,137 | $9,624 | $745 | $4,431 | $8,197 |
| 85+ | $16,545 | $12,520 | $13,402 | $12,192 | $1,670 | $19,679 | $1,695 | $429 | $4,431 | $15,078 |
| * Cost per incident case of disease. | | | | | | | | | | |
| ** Cost per prevalent case of disease. | | | | | | | | | | |
| *** Cost per person. | | | | | | | | | | |
| NB. Costs are in Australian dollars, adjusted to the year 2003. | | | | | | | | | | |

## Reference List

1. Keating CL, Dixon JB, Moodie ML, Peeters A, Playfair J, et al. (2009) Cost-efficacy of surgically induced weight loss for the management of type 2 diabetes: a randomized controlled trial. Diabetes Care 32: 580-584.

2. Buchwald H, Estok R, Fahrbach K, Banel D, Sledge I (2007) Trends in mortality in bariatric surgery: A systematic review and meta-analysis. Surgery 142: 621-635.

3. Keating CL, Dixon JB, Moodie ML, Peeters A, Bulfone L, et al. (2009) Cost-effectiveness of surgically induced weight loss for the management of type 2 diabetes: modeled lifetime analysis. Diabetes Care 32: 567-574.

4. Vos T, Carter R, Barendregt J, Mihalopoulos C, Veerman L, et al. (2010) Assessing Cost-Effectiveness in Prevention (ACE-Prevention). Centre for the Burden of Disease and Cost-Effectiveness, UQ School of Population Health.

5. Millar JA, Millward MJ (2007) Cost effectiveness of trastuzumab in the adjuvant treatment of early breast cancer: a lifetime model. Pharmacoeconomics 25: 429-442.

6. AIHW (2001) Disease costs and impacts study data. Canberra: AIHW.
